# Transesophageal echocardiography-associated tracheal microaspiration and ventilator-associated pneumonia in intubated critically ill patients: a multicenter prospective observational study

**DOI:** 10.1186/s13054-020-03380-w

**Published:** 2020-12-07

**Authors:** François Bagate, Anahita Rouzé, Farid Zerimech, Florence Boissier, Vincent Labbe, Keyvan Razazi, Guillaume Carteaux, Nicolas de Prost, Malika Balduyck, Patrice Maboudou, Saad Nseir, Armand Mekontso Dessap

**Affiliations:** 1 DHU A-TVB, Service de Médecine Intensive Réanimation, AP-HP, CHU Henri Mondor, 51, avenue du Mal de Lattre de Tassigny, 94010 Créteil, France; 2 Groupe de Recherche Clinique CARMAS, Faculté de Médecine, Université Paris Est Créteil, 94010 Créteil, France; 3 Service de Médecine Intensive Réanimation, CHU Lille, 59000 Lille, France; 4 Centre de Biologie Pathologie, CHU Lille, 59000 Lille, France; 5 Service de Médecine Intensive Réanimation, CHU Poitiers, Poitiers, France; 6 ALIVE Research Group, INSERM CIC 1402, University of Poitiers, Poitiers, France; 7 Service de Médecine Intensive Réanimation, Département Médico-Universitaire APPROCHES, Hôpital Tenon, Assistance Publique-Hôpitaux de Paris (AP-HP), Sorbonne Université, Paris, France; 8 Faculté de Pharmacie, Université de Lille, 59000 Lille, France; 9 Faculté de Médecine, Université de Lille, 59000 Lille, France; 10 INSERM U955, Institut Mondor de Recherche Biomédicale, 94010 Créteil, France

**Keywords:** Microaspiration, Transesophageal echocardiography, Pepsin, Salivary amylase

## Abstract

**Background:**

Microaspiration of gastric and oropharyngeal secretions is the main causative mechanism of ventilator-associated pneumonia (VAP). Transesophageal echocardiography (TEE) is a routine investigation tool in intensive care unit and could enhance microaspiration. This study aimed at evaluating the impact of TEE on microaspiration and VAP in intubated critically ill adult patients.

**Methods:**

It is a four-center prospective observational study. Microaspiration biomarkers (pepsin and salivary amylase) concentrations were quantitatively measured on tracheal aspirates drawn before and after TEE. The primary endpoint was the percentage of patients with TEE-associated microaspiration, defined as: (1) ≥ 50% increase in biomarker concentration between pre-TEE and post-TEE samples, and (2) a significant post-TEE biomarker concentration (> 200 μg/L for pepsin and/or > 1685 IU/L for salivary amylase). Secondary endpoints included the development of VAP within three days after TEE and the evolution of tracheal cuff pressure throughout TEE.

**Results:**

We enrolled 100 patients (35 females), with a median age of 64 (53–72) years. Of the 74 patients analyzed for biomarkers, 17 (23%) got TEE-associated microaspiration. However, overall, pepsin and salivary amylase levels were not significantly different between before and after TEE, with wide interindividual variability. VAP occurred in 19 patients (19%) within 3 days following TEE. VAP patients had a larger tracheal tube size and endured more attempts of TEE probe introduction than their counterparts but showed similar aspiration biomarker concentrations. TEE induced an increase in tracheal cuff pressure, especially during insertion and removal of the probe.

**Conclusions:**

We could not find any association between TEE-associated microaspiration and the development of VAP during the three days following TEE in intubated critically ill patients. However, our study cannot formally rule out a role for TEE because of the high rate of VAP observed after TEE and the limitations of our methods.

## Introduction

Ventilator-associated pneumonia (VAP) is the most common acquired infection in critically ill patients under mechanical ventilation [1], often associated with significant morbidity [2, 3]. VAP is mainly precipitated by microaspiration of contaminated gastric and oropharyngeal secretions [4]. Microaspiration is defined by leakage of oropharyngeal secretions accumulated upstream the tracheal cuff into the lower respiratory tract [5, 6]. The gold standard test for the diagnosis of microaspiration is using technetium 99 m [7]. However, applying this technique in intubated patients in the intensive care unit (ICU) is thwarted by the difficulty of transporting patients to the radiology department to avoid radioactivity in ICU [8]. Pepsin comes from pepsinogen and is secreted by the chief cells in the stomach, and amylase is a digestive enzyme, secreted by the salivary glands and the pancreas. Because they are not normally present in the respiratory tract, pepsin, and salivary amylase were proposed to diagnose microaspiration of gastric content and oropharyngeal secretions, respectively [9–12]. Their use in intubated critically ill patients is rapid, easy to perform in routine, cheap and only requires tracheal secretions.

Over the past decade, transesophageal echocardiography (TEE) has emerged as a common, minimally invasive, bedside examination in ICU [13], with a low complication rate in intubated patients [14, 15]. TEE-induced bacteremia is extremely rare; thus, TEE is not an indication for antibiotic prophylaxis [16]. Nevertheless, potential microaspiration associated with TEE has never been evaluated in intubated ICU patients. TEE could indirectly trigger microaspiration of oropharyngeal and gastric contents in mechanically ventilated patients via factors such as loss of integrity of the esophageal sphincter, gastroesophageal reflux, displacement of tracheal tube, and modification of tracheal cuff inflation.

The main objective of this study was to evaluate the role of TEE in triggering microaspiration of gastric contents and oropharyngeal secretions, and VAP in intubated critically ill patients.

## Methods

### Study design and participants

We performed a multicentric prospective observational study in four French medical ICUs of university hospitals between March 2017 and September 2018. Consecutive adult patients intubated and mechanically ventilated for more than 24 h prior to enrollment and who required TEE were included. Exclusion criteria were pregnancy, tracheostomy, and TEE contraindications. This study was conducted in compliance with the amended Declaration of Helsinki. The protocol was approved by the ethical committee CPP, Ile de-France III (EUDRACT number: 2016-A01488-43, approval number: S.C.3457). The protocol was considered as a component of standard care, and patient consent was waived. Written and oral information about the study was given to patients or families.

### Procedures and definitions

All included patients were subjected to endotracheal suction just before TEE and within the two hours after. For quantitative analyses, endotracheal aspirates were drawn without the addition of saline beforehand. The collected endotracheal aspirates were stored at − 20 °C in each center and sent to a central laboratory (Lille University Hospital) at the end of the study. All measurements of pepsin and amylase were performed by biologists who were blinded to the chronological status of TEE samples (before vs. after TEE). Pepsin was quantitatively measured by ELISA technique, and salivary amylase activity was calculated as the difference between total and pancreatic amylase activities [12, 17]. The tracheal cuff pressure was manually checked before and after TEE. For some patients included in the Henri Mondor center, Creteil, the tracheal cuff pressure was continuously and mechanically assessed from five minutes before TEE until five minutes after. For those patients, the tracheal cuff pressure signal was recorded using differential pressure transducer TSD160D (Biopac Systems, Goleta, CA, USA) connected to analog/numeric data acquisition system (MP150, Biopac systems, Goleta, CA, USA) and stored on a computer to be analyzed with AcqKnowledge software version 5.0 (Biopac systems, Goleta, CA, USA).

Microaspiration of gastric contents and oropharyngeal secretions is usually confirmed upon detecting significant pepsin (> 200 μg/L) [17] and salivary amylase (> 1685 IU/L) [12] concentrations in the tracheal secretions, respectively. TEE-associated microaspiration of gastric contents (or oropharyngeal secretions) was defined by the association of: (1) pepsin (or salivary amylase) concentration which is ≥ 50% higher in the post-TEE sample than in the pre-TEE sample and (2) a significant post-TEE concentration of pepsin of > 200 μg/L (or salivary amylase of > 1685 IU/L).

VAP diagnosis relied on clinical, radiological, and microbiological criteria. Namely, new and persistent infiltrate on chest X-rays (CXR) was associated with two of the following criteria: (1) turbid tracheal aspirates; (2) temperature > 38 °C or < 36 °C; and (3) peripheral leukocyte count > 10 G/L or < 1.5 G/L). All VAP diagnoses were documented by a positive microbiological sample of tracheal aspirate (≥ 10^5^ CFU/mL), protected telescopic catheter liquid (≥ 10^3^ CFU/mL), or bronchoalveolar lavage (≥ 10^4^ CFU/mL). Tracheobronchial colonization was confirmed by a positive (≥ 10^5^ CFU/ml) tracheal aspirate without CXR signs of VAP [18]. The participating ICUs management fulfilled the VAP-prevention guidelines [18, 19].

### Data collection

All data were prospectively collected starting with the inclusion data: age, gender, body mass index, simplified acute physiology score II (SAPS II) at ICU admission [20], comorbidities, history of acute respiratory distress syndrome, shock, and VAP prior to TEE, date and cause of intubation, tracheal tube characteristics (type, diameter, position), Sequential Organ Failure Assessment (SOFA) score, Richmond Agitation and Sedation Scale (RASS), duration of mechanical ventilation prior to TEE, time between last oral decontamination and TEE, ventilator parameters, tracheal cuff pressure before and after TEE, gastric tube and enteral feeding management, evaluation of residual gastric volume, concomitant treatments, probe type and introduction (duration, number of attempts, method, patient position), TEE characteristics (date, duration, indication, use of transgastric view), and complications. The following data were collected during ICU stay: length of stay, mechanical ventilation duration, VAP, and mortality.

### Outcomes

The primary endpoint of this study was the percentage of patients with TEE-associated microaspiration of gastric contents and/or oropharyngeal secretions. The secondary outcomes were the percentage of patients who developed VAP within three days after TEE and the evolution of tracheal cuff pressure throughout TEE procedure.

### Statistical analysis

Statistical analysis was performed using JMP software (version 9; SAS Institute Inc, Cary, NC) and GraphPad Prism 5 software (GraphPad Software Inc., La Jolla, CA, USA). The number of patients required to assess the incidence rate of microaspiration during TEE was estimated at 75, considering a theoretical prevalence of 75% (previous studies reported the presence of microaspiration at baseline in at least 50% of intubated patients) [12, 21, 22], a precision of ± 10%, a confidence interval of 95%, and a type I error rate of 5%. We anticipated a 25% failure rate for sample processing and analysis and decided to include a total of 100 patients.

Normality of variables was evaluated by Shapiro–Wilk test. Continuous variables were expressed as mean (± standard deviation) or median (first quartile–third quartile) according to their Gaussian or non-Gaussian distribution, respectively. We compared patients who developed VAP within the three days following TEE with their counterparts using Student *t* test for Gaussian continuous variables, Mann–Whitney test for non-Gaussian continuous variables, and Chi-square or Fisher exact tests for categorical variables, as appropriate. We compared concentrations of pepsin and salivary amylase before and after TEE using paired Wilcoxon test. We evaluated the change in tracheal cuff pressure throughout TEE procedure using one-way ANOVA and Dunnett multiple comparison test. For all tests, a two-tailed *P* < 0.05 was considered statistically significant.

## Results

### The study population

**Fig. 1:**
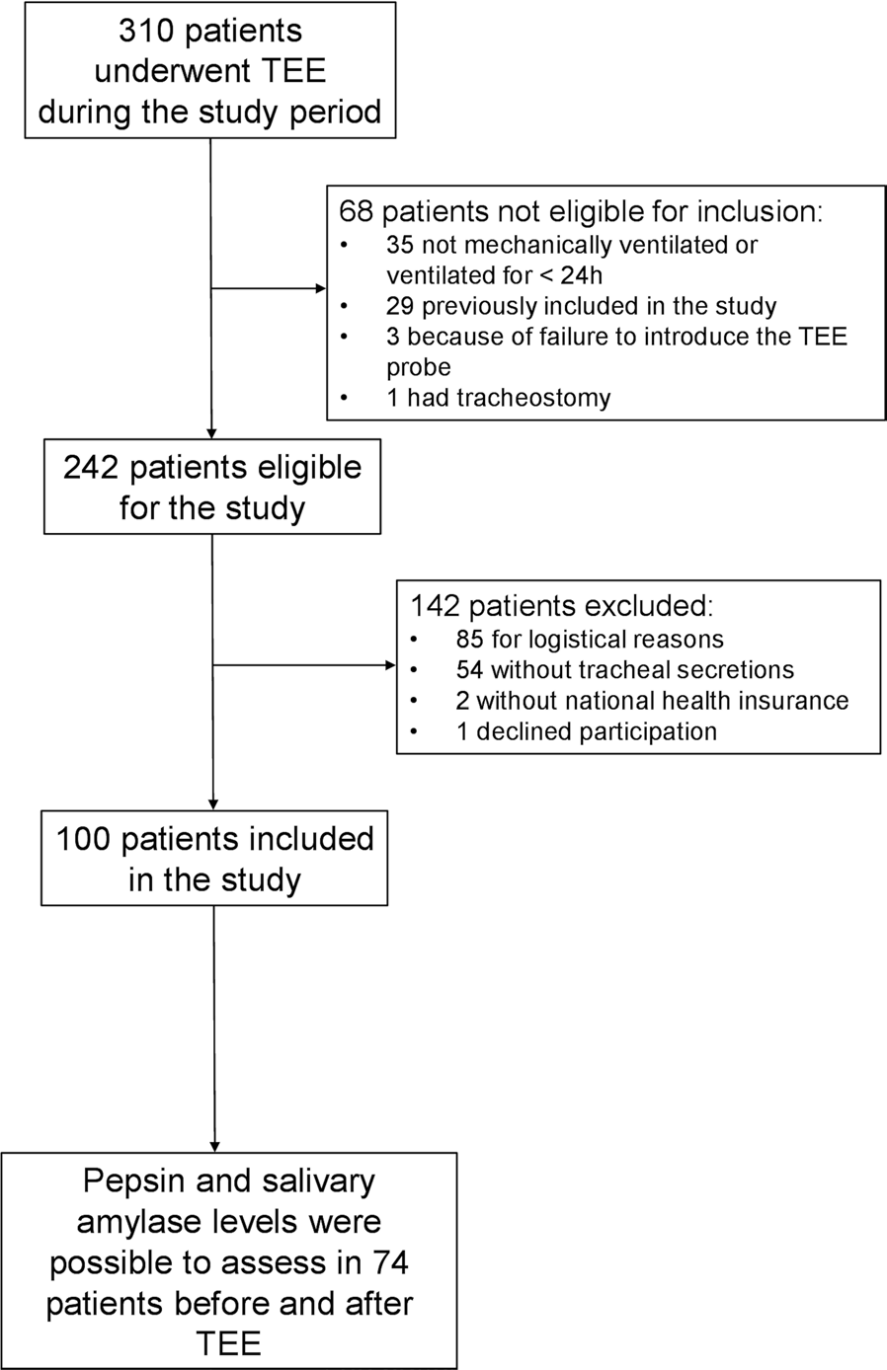
Flowchart. *TEE* transesophageal echocardiography

A total of 310 patients who underwent TEE were screened during the study period in the participating centers, of whom 242 met the eligibility criteria; however, only 100 patients (35 females) were retained in this study (Fig. 1), with a median age of 64 (53–72) years. The majority of eligible patients were excluded for logistical reasons (absence of the investigator when TEE was performed, at night and on weekends) or because of lack of sufficient tracheal secretions. During TEE examination, most patients were already sedated (93%) and sedation was increased in many of them (62%), but only few (*n* = 12, 12%) received additional neuromuscular blocking agent.

Altogether, 19/100 patients (19%) were diagnosed with VAP within three days after TEE. Patients’ characteristics at baseline and throughout TEE procedure with comparison between those who developed VAP and those who did not are shown in Tables 1 and 2, respectively. VAP patients had a larger tracheal tube size, endured more attempts of TEE probe introduction, and were more often on anticoagulants than no-VAP patients. TEE complications were scarce and similar in both groups. Among the 19 VAP episodes, three had no bacteriological documentation and six were polymicrobial. The causative microorganisms identified were *Pseudomonas aeruginosa* (seven cases), *Klebsiella pneumoniae* (six cases), *Staphylococcus aureus* (three cases), *Enterobacter cloacae* (three cases), *Stenotrophomonas maltophilia* (three cases*), Escherichia coli* (two cases) and *Proteus mirabilis* (one case).

**Table 1:** Patients’ characteristics at ICU admission and at enrollment, according to VAP occurrence within three days after TEE

Variables	Total (*n* = 100)	VAP (*n* = 19)	No VAP (*n* = 81)	*p* value
Age (years)	64 (53–72)	63 (58–68)	64 (53–72)	0.94
Female gender	35 (35%)	4 (21.1%)	31 (38.3%)	0.16
Body mass index (Kg/m^2^)	28 (24–33)	28 (24–32)	27 (24–33)	0.93
SAPS-II at admission	52.6 (± 17.3)	49.7 (± 15.3)	53.2 (± 17.7)	0.41
*Comorbidities*				
COPD	8 (8%)	3 (15.8%)	5 (6.2%)	0.17
Diabetes mellitus	33 (33%)	9 (47.4%)	24 (29.6%)	0.14
Swallowing disorder	5 (5%)	2 (10.5%)	3 (3.7%)	0.24
ENT or esophageal surgery	2 (2%)	1 (5.3%)	1 (1.2%)	0.35
Cirrhosis	1 (1%)	0	1 (1.2%)	> 0.99
Immunodeficiency	21 (21%)	4 (21.1%)	17 (21.0%)	> 0.99
Steroid therapy	17 (17%)	3 (15.8%)	14 (17.3%)	> 0.99
*Complications before TEE*				
Acute respiratory distress syndrome	38 (38%)	8 (42.1%)	30 (37.0%)	0.68
Shock	74 (74%)	13 (68.4%)	61 (75.3%)	0.54
VAP	21 (21%)	6 (31.6%)	15 (18.5%)	0.21
*Endotracheal intubation characteristics*				
Reason for mechanical ventilation				0.83
Acute respiratory distress	47 (47%)	10 (52.6%)	37 (45.7%)	
Shock	17 (17%)	3 (15.8%)	14 (17.3%)	
Coma	25 (25%)	5 (26.3%)	20 (24.7%)	
Others	11 (11%)	1 (5.3%)	10 (12.4%)	
ETT type				0.13
Standard	58 (58.6%)	7 (38.9%)	51 (63.0%)	
Subglottic secretion drainage tube	40 (40.4%)	11 (61.1%)	29 (35.8%)	
Others	1 (1.0%)	0	1 (1.2%)	
Tracheal tube size (mm)	7.5 (7.5–8)	8.0 (7.5–8)	7.5 (7.5–8)	0.02
Distance from ETT tip to the carina (cm)	3.7 (2.5–5.0)	3.1 (2.4–4.9)	3.7 (2.7–5.3)	0.50
*At enrollment*				
SOFA	10 (5–13)	10 (5–13)	10 (5–13)	0.96
Duration of prior mechanical ventilation (d)	3 (1–8)	5 (1–9)	2 (1–8)	0.35
RASS	− 4 (− 5 to − 2)	− 5 (− 5 to − 2)	− 4 (− 5 to − 2)	0.59
Oral decontamination-to-TEE time (h)	3 (2–6)	4 (1–6)	3 (2–6)	0.95
*Ventilation parameters*				
Mode of ventilation				0.88
Assist-control ventilation	74 (74%)	14 (73.7%)	60 (74.1%)	
Pressure support ventilation	25 (25%)	5 (26.3%)	20 (24.7%)	
Others	1 (1%)	0	1 (1.2%)	
Tidal volume (mL)	400 (370–450)	415 (365–450)	400 (367–445)	0.68
Respiratory rate (/min)	25 (22–32)	26 (22–33)	25 (21–31)	0.36
Positive end-expiratory pressure (cmH_2_O)	6 (5–10)	5 (5–8)	6 (5–10)	0.19
Peak pressure (cmH_2_O)	32 (± 10)	32 (± 9)	32 (± 10)	0.91
Plateau pressure (cmH_2_O)	21 (± 6)	21 (± 6)	21 (± 6)	0.92

Values are expressed as mean (± SD) or median (IQR) as appropriate. *VAP* ventilator-associated pneumonia, *TEE* transesophageal echocardiography, *SAPS II* Simplified Acute Physiology Score II, *COPD* chronic obstructive pulmonary disease, *ENT* ear nose throat, *ETT* endotracheal tube, *SOFA score* Sequential Organ Failure Assessment, *RASS* Richmond Agitation and Sedation Scale

**Table 2:** Patients’ characteristics before, after, and at TEE, according to VAP occurrence within three days after TEE

Variables	Total (*n* = 100)	VAP (*n* = 19)	No VAP (*n* = 81)	*p* value
*Tracheal cuff pressure management (cmH*_*2*_*O)*				
Mechanical device controlling tracheal cuff pressure	6 (6%)	3 (15.8%)	3 (3.7%)	0.08
Tracheal cuff pressure before TEE	30 (25–30)	30 (25–30)	30 (25–30)	0.87
Tracheal cuff pressure after TEE	25 (20–30)	30 (23–41)	25 (20–30)	0.07
*Enteral feeding*				
Use of nasogastric tube	28 (28.3%)	2 (10.5%)	26 (32.5%)	0.09
Use of orogastric tube	67 (67.7%)	16 (84.2%)	51 (63.8%)	0.11
Enteral feeding before TEE	64 (64%)	13 (68.4%)	51 (63.0%)	0.66
Discontinuing enteral feeding for TEE	24 (24%)	3 (15.8%)	21 (25.9%)	0.55
Evaluation of residual gastric volume	9 (9%)	1 (5.3%)	8 (9.9%)	> 0.99
Gastric tube removal for TEE	7 (7.7%)	0	7 (8.9%)	0.60
Use of transgastric view	78 (78%)	17 (89.5%)	61 (75.3%)	0.23
*Concurrent treatment*				
Use of sedation	93 (93%)	18 (94.7%)	75 (92.6%)	> 0.99
Increase in sedation level	62 (62%)	13 (68.4%)	49 (60.5%)	0.61
Use of neuromuscular blocking agent	40 (40%)	8 (42.1%)	32 (39.5%)	0.84
Adding neuromuscular blocking agent for TEE	12 (12%)	2 (10.5%)	10 (12.4%)	> 0.99
Catecholamines	52 (52%)	11 (57.9%)	41 (50.6%)	0.62
Antibiotic therapy	86 (86%)	16 (84.2%)	70 (86.4%)	0.73
Use of proton pump inhibitor	64 (64.6%)	15 (78.9%)	49 (61.3%)	0.19
Anticoagulants	28 (28%)	9 (47.4%)	19 (23.5%)	0.04
*TEE characteristics*				
Indications				0.20
Hemodynamic evaluation	22 (22%)	6 (31.6%)	16 (19.8%)	
Severe hypoxemia	13 (13%)	2 (10.5%)	11 (13.6%)	
Suspected endocarditis	42 (42%)	6 (31.6%)	36 (44.4%)	
Before cardioversion	6 (6%)	3 (15.8%)	3 (3.7%)	
Others	17 (17%)	2 (10.5%)	15 (18.5%)	
Probe introduction				
Mandible elevation	47 (49.0%)	13 (68.4%)	34 (44.2%)	0.06
Neck anteflexion	65 (67.7%)	15 (79.0%)	50 (64.9%)	0.29
Use of laryngoscope	15 (15.5%)	1 (5.3%)	14 (18.0%)	0.29
Number of attempts	2 (1–2)	2 (2–3)	1 (1–2)	0.03
More than one attempt	53 (55.2%)	15 (79.0%)	38 (49.4%)	0.02
Introduction duration (min)	1 (1–2)	2 (1–3)	1 (1–2)	0.13
Patient position				0.94
Completely supine position (0°)	17 (17%)	3 (15.8%)	14 (17.3%)	
Semi-recumbent position (45°)	79 (79%)	15 (79.0%)	64 (79.0%)	
Prone position	4 (4%)	1 (5.3%)	3 (3.7%)	
TEE duration (min)	26 (18–35)	30 (22–33)	25 (17–35)	0.16
Total duration including introduction (min)	28 (21–36)	31 (23–34)	27 (21–37)	0.20
*TEE-associated complications*				
Profound desaturation (< 80%)	1 (1%)	0	1 (1.2%)	> 0.99
Arterial hypotension	13 (13%)	0	13 (16.1%)	0.12
Arterial hypertension	1 (1%)	0	1 (1.2%)	> 0.99
Minor bleeding	11 (11%)	4 (21.1%)	7 (8.6%)	0.21
Major bleeding	0	0	0	-
Vomiting	2 (2%)	0	2 (2.5%)	> 0.99

Values are expressed as mean (± SD) or median (IQR) as appropriate. *TEE* transesophageal echocardiography, *VAP* ventilator-associated pneumonia

### Microaspiration

**Fig. 2:**
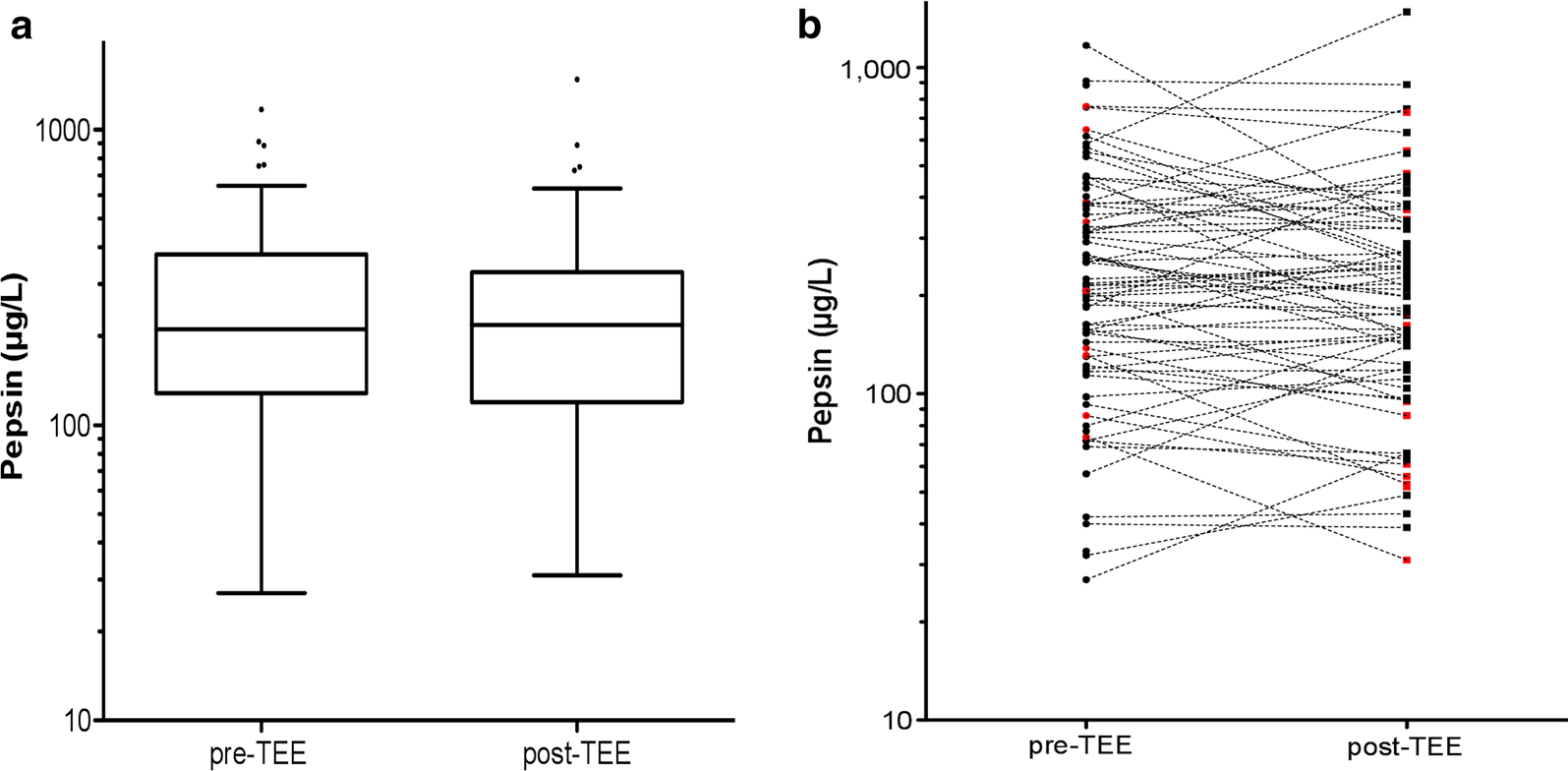
Pepsin variation before and after TEE with Box-and-Whisker plots (**a**) and individual values (**b**; VAP patients are in red). *TEE* transesophageal echocardiography, *VAP* ventilator-associated pneumonia

**Fig. 3:**
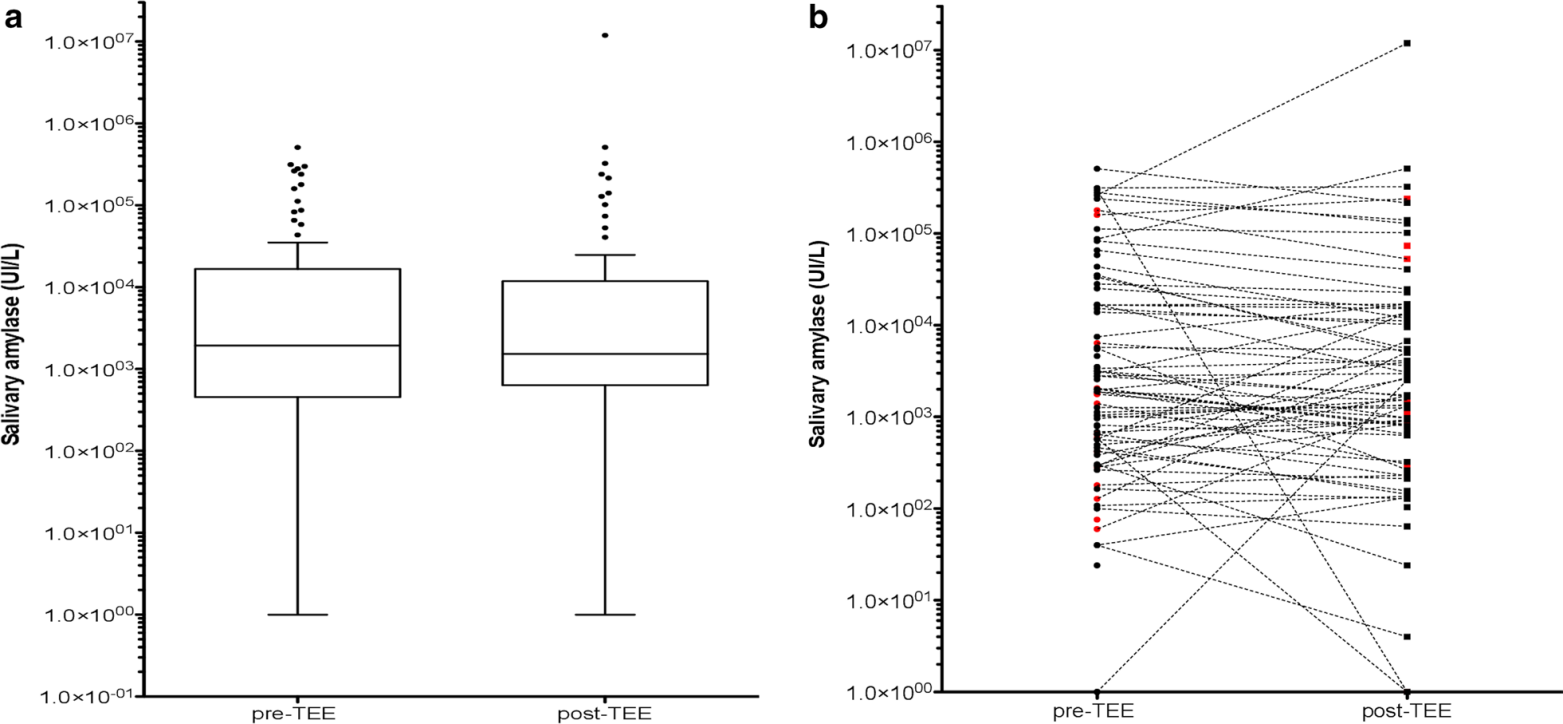
Salivary amylase variation before and after TEE with Box-and-Whisker plots (**a**) and individual values (**b**; VAP patients are in red). *TEE* transesophageal echocardiography, *VAP* ventilator-associated pneumonia

It was possible to assess pepsin and salivary amylase concentrations (sufficient amount of tracheal suction) in 82 patients before TEE, 83 patients after TEE, and 74 patients for both time points (Fig. 1). We detected 17/74 patients with TEE-associated microaspiration (prevalence of 23%, 95% confidence interval 15–34%), and this prevalence did not differ between the four participating centers. The concentrations of pepsin and salivary amylase were not different between VAP and no-VAP patients (Table 3). Moreover, median pepsin and salivary amylase levels were not significantly different before and after TEE (Figs. 2 and 3). No association was found between the occurrence of VAP within three days of TEE and TEE-associated microaspiration (Table 3). A sensitivity analysis assessing patients who developed VAP within 5 days following TEE (22/100, 22%) found similar results (Additional file 1: Table S1).

**Table 3:** Microaspiration indicators and outcomes stratified by VAP incidence within 3 days after TEE

Variables	*n*	Total (*n* = 100)	VAP (*n* = 19)	No VAP (*n* = 81)	*p* value
Pre-TEE pepsin (ng/mL)	82	211 (128–379)	154 (86–337)	216 (130–380)	0.49
Pre-TEE pepsin > 200 μg/L	82	47 (57.3%)	7 (46.7%)	40 (59.7%)	0.36
Post-TEE pepsin (ng/mL)	83	218 (120–329)	162 (56–368)	229 (140–323)	0.37
Post-TEE pepsin > 200 μg/L	83	44 (53.0%)	6 (40.0%)	38 (55.9%)	0.26
Pre-TEE salivary amylase (IU/L)	82	1932 (454–16700)	648 (180–2052)	2792 (568–25220)	0.10
Pre-TEE salivary amylase > 1685 IU/L	82	45 (54.9%)	6 (40.0%)	39 (58.2%)	0.20
Post-TEE salivary amylase (IU/L)	83	1532 (632–11820)	1096 (304–5108)	1710 (636–11889)	0.58
Post-TEE salivary amylase > 1685 IU/L	83	40 (48.2%)	5 (33.3%)	35 (51.5%)	0.20
TEE-associated pepsin absolute variation	70	− 5 (− 59 to 32)	− 31 (− 72 to 15)	0 (− 59 to 35)	0.42
TEE-associated salivary amylase absolute variation	70	− 216 (− 2760 to 564)	− 502 (− 1195 to 1095)	− 186 (− 5095 to 468)	0.86
TEE-associated microaspiration	74	17 (23.0%)	3 (23.1%)	14 (22.9%)	> 0.99
*Other outcomes*					
Successful extubation	99	62 (62.6%)	10 (52.6%)	52 (65.0%)	0.43
MV duration after TEE (d)	99	8 (3–17)	10(6–18)	8 (3–17)	0.37
Extubation within three days after TEE	99	26 (26.3%)	3 (15.8%)	23 (28.8%)	0.39
MV duration (d)	99	14 (7–27)	12 (8–33)	14 (6–27)	0.70
ICU length of stay (d)	99	20 (9–32)	17 (10–37)	21 (9–32)	0.91
ICU mortality	99	36 (36.4%)	8 (42.1%)	28 (35.0%)	0.60

Values are expressed as mean (± SD) or median (IQR) as appropriate

*TEE* transesophageal echocardiography, *VAP* ventilator-associated pneumonia, *MV* mechanical ventilation, *ICU* intensive care unit

### Continuous monitoring of tracheal cuff pressure

**Fig. 4:**
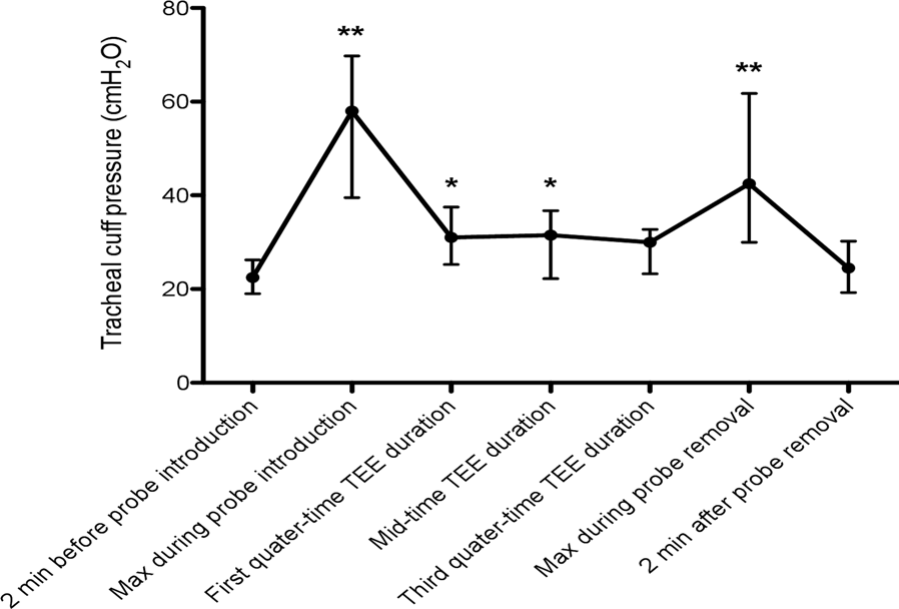
Evolution of tracheal cuff pressure throughout TEE procedure. * and **denote significant difference as compared with baseline, i.e., tracheal cuff pressure 2 min before probe introduction, with a p value < 0.05 and < 0.01, respectively.

Continuous monitoring of tracheal cuff pressure throughout TEE process was performed in 20 patients, of whom six had TEE-associated microaspiration and three had VAP. Overall, as compared with baseline (2 min before TEE start), TEE induced an important increase in tracheal cuff pressure, especially during insertion and removal of the TEE probe (Fig. 4).

## Discussion

To the best of our knowledge, this is the first study conducted to evaluate the impact of performing TEE on the occurrence of microaspiration and VAP in intubated critically ill patients. Although a substantial number of patients could be characterized as having TEE-associated microaspirations (23%), according to an ad hoc definition, the changes in pepsin and salivary amylase levels throughout TEE process showed huge interindividual variability. We detected no association between TEE-associated microaspiration and the development of VAP during the three days following TEE. However, because of the high rate of VAP observed after TEE and the limitations of our methods, our findings cannot formally rule out a role for TEE in the occurrence of VAP. TEE generated a transient variation of tracheal cuff pressure, especially upon inserting and removing the TEE probe.

Microaspiration is a well-known causative factor of VAP [23]. Pepsin and salivary amylase are reliable markers of microaspiration and are tightly linked to the development of VAP [21, 24, 25]. These markers have been used as surrogates in studies evaluating the efficacy of various devices in preventing VAP, as tracheal tubes [26], subglottic secretion drainage systems [27], and mechanical devices controlling tracheal cuff pressure [17]. In such studies, microaspiration assessment relied on several tracheal aspirates drawn over a wide timeframe (1 or 2 days), and its definition considered the percentage of tracheal aspirates with higher levels of pepsin (> 200 μg/L) and/or salivary amylase (> 1685 IU/L). For us, it was not possible to evaluate TEE-associated microaspiration using the same approach given the limited number of tracheal aspirates available in our protocol (only two/patient). Of more, we relied on commonly reported thresholds for salivary amylase and pepsin [8, 12, 26].

The continuous monitoring of tracheal cuff pressure throughout TEE procedure showed significant elevation of cuff pressure, especially during insertion and removal of the TEE probe. Persistent underinflation (< 20 cmH_2_O) of the tracheal cuff was shown as an independent risk factor for microaspiration and VAP [28], whereas cuff leakage was inversely correlated with cuff pressure [29]. Hypothesizing that acute variations of tracheal cuff pressure during TEE might be associated with microaspiration and VAP warrants further research.

The relatively high rate of VAP found in this study can be reasonably attributed to the severe cases we included. Of note, 38% of patients presented with acute respiratory distress syndrome. However, we cannot formally exclude a role of microaspiration in this high rate. The fact that patients who caught VAP had their tracheal tubes larger than those used in patients who did not may suggest more leaks occurring in the former group. Moreover, patients who caught VAP were more often on anticoagulant, a therapy that has potential anti-inflammatory effects beyond anticoagulation, and which may be beneficial in acute respiratory distress syndrome [30]. For instance, nebulized heparin was proposed for lung injury but with contradictory results [31] and was not effective in preventing VAP [32].

We did not identify any dreaded clinical complication associated with TEE neither did TEE significantly impact salivary amylase and pepsin concentrations. However, the substantial levels of pepsin and amylase observed in some patients and the fact that VAP patients had endured more attempts of TEE probe introduction might represent a good incentive to install some VAP prevention measures before and/or during TEE. Such measures may involve deep oropharyngeal suctioning [33], subglottic suctioning [34], semi-recumbent positioning [35], continuous control of tracheal cuff pressure [17], or using higher PEEP levels [29].

This multicenter study was conducted in four tertiary university ICUs where TEE is routinely used in intubated critically ill patients. The major strengths of the study are the comprehensive search for risk factors for microaspiration, its prospective design, the combined use of salivary amylase and pepsin for microaspiration documentation, and the continuous assessment of tracheal cuff pressure to scrutinize VAP pathophysiology. Our study has several limitations. First, the cohort included a relatively small number of patients with no control arm. Second, pepsin and salivary amylase and tracheal cuff pressure continuous monitoring were not assessed in all patients. Third, the definition of TEE-associated microaspiration may be questionable, as previously discussed. It used a single assessment of biomarkers and an arbitrary cutoff. We did not correct for baseline concentration of biomarkers in the digestive tract, but these biomarkers are not normally found in the respiratory tract and previous studies did not use such corrections. Fourth, the use of three days as a cutoff point to define VAP after TEE is also questionable, but results were similar upon using a five-day cutoff point. Fifth, we focused on direct microaspiration during TEE and did not assess other mechanisms that may cause pneumonia, as dysphagia or swallowing dysfunction [36]. Eventually, we did not assess the change in tracheal bacterial colonization. The amount of bacterial inoculum could be used as a closer surrogate for VAP [37]. Sixth, VAP would have been more relevant as a primary endpoint from a clinical point of view. However, if TEE has a potential impact on VAP, it is likely to be small given the multiple factors influencing VAP occurrence. We therefore used microaspiration as the primary endpoint because microaspiration is considered as the main mechanism of VAP. Lastly, the limitations of the methods used to identify TEE-associated microaspiration and the high rate of VAP observed after TEE cannot allow ruling out a role for TEE.

## Conclusion

In this multicenter prospective observational study, we detected no association between TEE-associated microaspiration and the development of VAP during the three days following TEE. However, because of the high level of VAP observed after TEE and the limitations of the methods used, our findings cannot allow formally ruling out a role for TEE in the occurrence of VAP.

## Supplementary information

**Additional file 1: Table S1**. Microaspiration indicators and outcomes stratified by VAP incidence within 5 days after TEE.

## Data Availability

All data generated and analyzed during the study are included in the published article and can be shared upon request. All authors helped to revise the draft of the manuscript. All authors read and approved the final manuscript.

## Abbreviations

VAP: Ventilator-associated pneumonia
ICU: Intensive care unit
TEE: Transesophageal echocardiography
CXR: Chest X-rays
SAPS II: Simplified acute physiology score II
SOFA: Sequential organ failure assessment
RASS: Richmond agitation and sedation scale
