# Supplementary material for: Transesophageal echocardiography-associated tracheal microaspiration and ventilator-associated pneumonia in intubated critically ill patients: a multicenter prospective observational study
Source: Crit Care. 2020 Dec 7;24:679. doi: 10.1186/s13054-020-03380-w (PMC7719845; doi:10.1186/s13054-020-03380-w)
Supplement: Supplementary file 1 — Additional file 1: Table S1. Microaspiration indicators and outcomes stratified by VAP incidence within 5 days after TEE. [file 13054_2020_3380_MOESM1_ESM.docx]

| **TABLE S1** Microaspiration indicators and outcomes stratified by VAP incidence within five days after TEE | | | | | |
| --- | --- | --- | --- | --- | --- |
| **Variables** | **n** | **Total**  **(n=100)** | **VAP**  **(n=22)** | **No VAP**  **(n=78)** | ***p* value** |
| Pre-TEE pepsin (ng/mL) | 82 | 211 (128-379) | 181 (97-331) | 215 (128-382) | 0.53 |
| Pre-TEE pepsin >200 μg/L | 82 | 47 (57.3%) | 8 (50%) | 39 (59.1%) | 0.51 |
| Post-TEE pepsin (ng/mL) | 83 | 218 (120-329) | 177 (59-368) | 223 (140-324) | 0.47 |
| Post-TEE pepsin >200 μg/L | 83 | 44 (53.0%) | 8 (47.1%) | 36 (54.6%) | 0.58 |
| Pre-TEE salivary amylase (IU/L) | 82 | 1,932 (454-16,700) | 1,024 (206-4,710) | 2,684 (550-25,958) | 0.15 |
| Pre-TEE salivary amylase >1,685 IU/L | 82 | 45 (54.9%) | 7 (43.8%) | 38 (57.6%) | 0.32 |
| Post-TEE salivary amylase (IU/L) | 83 | 1,532 (632-11,820) | 1,096 (284-8,464) | 1,710 (644-12,328) | 0.57 |
| Post-TEE salivary amylase >1,685 IU/L | 83 | 40 (48.2%) | 6 (35.3%) | 34 (51.5%) | 0.23 |
| TEE-associated pepsin absolute variation | 70 | -5 (-59-32) | -30 (-65-20) | -1 (-60-37) | 0.54 |
| TEE-associated salivary amylase absolute variation | 70 | -216 (-2,760-564) | -583 (-2,006-918) | -184 (-3,884-500) | 0.89 |
| TEE- associated microaspiration | 74 | 17 (23.0%) | 3 (21.4%) | 14 (23.3%) | >0.99 |
| **Other outcomes** |  |  |  |  |  |
| Successful extubation | 99 | 62 (62.6%) | 11 (50%) | 51 (66.2%) | 0.17 |
| MV duration after TEE (d) | 99 | 8 (3-17) | 8 (6-14) | 8 (3-17) | 0.45 |
| Extubation within three days after TEE | 99 | 26 (26.3%) | 3 (13.6%) | 23 (29.9%) | 0.17 |
| MV duration (d) | 99 | 14 (7-27) | 11 (8-23) | 14 (6-28) | 0.90 |
| ICU length of stay (d) | 99 | 20 (9-32) | 15 (9-35) | 21 (10-32) | 0.54 |
| ICU mortality | 99 | 36 (36.4%) | 9 (40.9%) | 27 (35.1%) | 0.62 |
| Values are expressed as mean (±SD) or median (IQR) as appropriate. TEE: Transesophageal echocardiography, VAP: ventilator-associated pneumonia, MV: mechanical ventilation, ICU: intensive care unit. | | | | | |
